# Seasonality and Trend Forecasting of Tuberculosis Prevalence Data in Eastern Cape, South Africa, Using a Hybrid Model

**DOI:** 10.3390/ijerph13080757

**Published:** 2016-07-26

**Authors:** Adeboye Azeez, Davies Obaromi, Akinwumi Odeyemi, James Ndege, Ruffin Muntabayi

**Affiliations:** 1 Biostatistics and Epidemiology Research Group, Department of Statistics, University of Fort Hare, PMB X1314, Alice 5700, South Africa; daviesobaromi@gmail.com; 2 Department of Statistics, University of Fort Hare, PMB X1314, Alice 5700, South Africa; aodeyemi@ufh.ac.za (A.O.); jndege@ufh.ac.za (J.N.); mmutambayi@ufh.ac.za (R.M.)

**Keywords:** autocorrelation, co-infection, neutral-network, non-seasonality, prediction

## Abstract

*Background*: Tuberculosis (TB) is a deadly infectious disease caused by *Mycobacteria tuberculosis*. Tuberculosis as a chronic and highly infectious disease is prevalent in almost every part of the globe. More than 95% of TB mortality occurs in low/middle income countries. In 2014, approximately 10 million people were diagnosed with active TB and two million died from the disease. In this study, our aim is to compare the predictive powers of the seasonal autoregressive integrated moving average (SARIMA) and neural network auto-regression (SARIMA-NNAR) models of TB incidence and analyse its seasonality in South Africa. *Methods*: TB incidence cases data from January 2010 to December 2015 were extracted from the Eastern Cape Health facility report of the electronic Tuberculosis Register (ERT.Net). A SARIMA model and a combined model of SARIMA model and a neural network auto-regression (SARIMA-NNAR) model were used in analysing and predicting the TB data from 2010 to 2015. Simulation performance parameters of mean square error (MSE), root mean square error (RMSE), mean absolute error (MAE), mean percent error (MPE), mean absolute scaled error (MASE) and mean absolute percentage error (MAPE) were applied to assess the better performance of prediction between the models. *Results*: Though practically, both models could predict TB incidence, the combined model displayed better performance. For the combined model, the Akaike information criterion (AIC), second-order AIC (AICc) and Bayesian information criterion (BIC) are 288.56, 308.31 and 299.09 respectively, which were lower than the SARIMA model with corresponding values of 329.02, 327.20 and 341.99, respectively. The seasonality trend of TB incidence was forecast to have a slightly increased seasonal TB incidence trend from the SARIMA-NNAR model compared to the single model. *Conclusions*: The combined model indicated a better TB incidence forecasting with a lower AICc. The model also indicates the need for resolute intervention to reduce infectious disease transmission with co-infection with HIV and other concomitant diseases, and also at festival peak periods.

## 1. Introduction

Tuberculosis (TB) is a deadly infectious disease caused by *Mycobacteria tuberculosis*. TB incidence occurs in every part of the world. More than 95% of TB mortality occurs in low/middle income countries, and it is among the five leading causes of mortality in women aged 15 to 44 [1,2]. In 2014, approximately ten million people were diagnosed with active TB and two million died from the disease [3]. The highest incidence of new TB cases occurred in the Western Pacific Regions and South-East Asia with a record of over 58% of new cases worldwide. However, Africa bears the highest severe burden, with estimated 281 TB cases per 100,000 population in 2014. Almost 80% of reported cases of TB occurred in 22 countries. The six countries distinguished to have the highest incidence in 2014 were India, Indonesia, Nigeria, Pakistan, China and South Africa [4].

South Africa is one of the highest-disease burdened countries in the world for TB and TB/HIV co-infection. The World Health Organization (WHO) cited 22 high-burden countries (HBCs), which account for about 81% of TB incidence cases globally. South Africa is the third highest among the HBCs reported to have TB incidence cases and the fifth highest number of estimated prevalent cases. It also has the largest numbers of TB and HIV co-infections and the second-largest incidence of multidrug-resistant (MDR) TB cases [4].

In 1994, after South Africa became a democratic system, the National Tuberculosis Programme (NTP) was established to tackle the challenges of providing TB services to an insubstantial primary healthcare system and the face the advent of the HIV co-infection epidemic, which advanced to increase the number of TB cases fourfold between 1994 and 2012 [5]. The burden of rising MDR-TB and extensively drug-resistant (XDR) TB rates in 2006 added even more burden to a stressed health services system. South Africa’s rate of treatment success among newly diagnosed smear-positive and smear-negative as well as extra-pulmonary TB patients has increased to 79%, 76% and 50% respectively. This was achieved largely as a result of an improvement in TB cure rates and reduction in the rate of treatment non-compliance due to the establishment of community-based follow-up teams [4]. The rate of treatment success among relapse cases remains however poor at 66.3% [6]. It is worrisome that up to 25% cases of sputum smear-positive TB are lost to follow-up before initiating treatment, which contributes to transmission progression and increased death risks [7]. The death rate still remains high, even at the end of TB treatment, which may probably be due to HIV co-infection [8].

There were some retrospective studies on the seasonality and trend analysis of TB data to describe the trends of TB incidence [9,10,11,12]. In many countries, various models have been used to forecast TB in order to figure out the trends and predict the root cause of the TB incidence epidemic [13,14,15]. Though there are a lot of nations that keep TB information records from population-based studies but there has not been a national survey of the TB prevalence epidemic in any country.

The purpose of this study was to compare a hybrid model to forecast the TB incidence epidemic with an existing model and to assess the model seasonality trends in South Africa. Many models such as Markov chain models [16], autoregressive integrated moving average class models (ARIMA), general regression models, Grey models [17] and neural networks [18] have been proposed, which can be used to forecast infectious diseases. For better forecasting performance, a comparison of two models to forecast infectious disease was studied. The results from this study will be helpful to predict future TB incidence epidemics and optimize TB control and intervention using the predictions as reference information.

## 2. Methods

### 2.1. Study Setting

**Figure 1:**
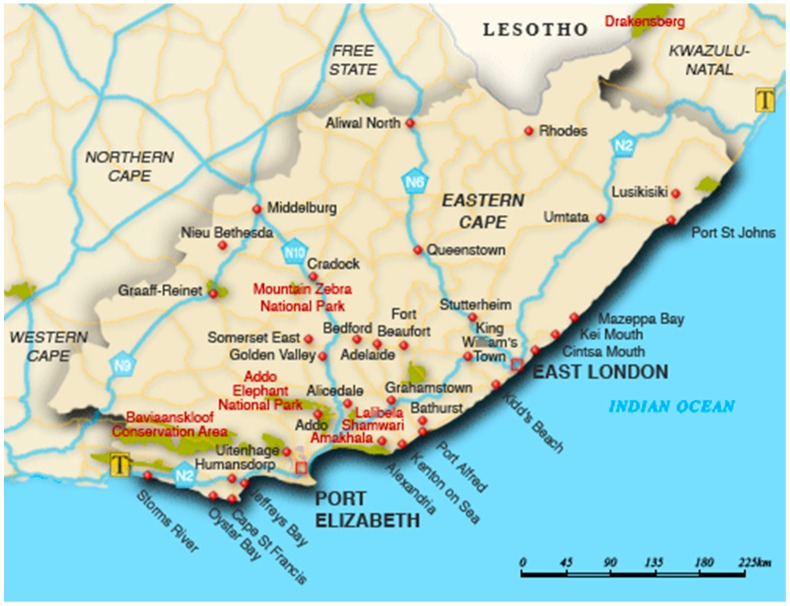
Map showing Eastern Cape Province, South Africa. Map data ©2016 AfriGIS (Pty) Ltd., Google.

Eastern Cape (EC) is the second largest province in South Africa, with an area about 170,000 square kilometres, which is almost the size of Uruguay (Figure 1). It occupies about 13.9% of South Africa’s entire land area and the entire population is about 6.5 million persons, which makes it the third largest population in South Africa. The EC racial population distribution is 86.3% black, 8.3% coloured, 4.7% white and 0.4% Indian/Asian. The proportion of the latter group has increased with most migrants coming from Sub-Saharan Africa, Indian and Asia. The capital city is Bhisho with the two most populous cities being East London and Port Elizabeth. EC is located on the South Eastern coast with many naturally beautiful spots, especially the rocky cliffs, oceans and thick green scrublands known as the wild coastline.

### 2.2. Data Collection

We studied retrospective data of all confirmed TB cases reported to the Eastern Cape Department of Health from 2010 to 2015 recorded on the noticeable infectious diseases occurrence data form monthly and yearly. The data occurrence and death rates of every single notifiable infection were mainly pulled together from all the TB hospitals in the province. Laboratory confirmation was based on Smear Positive Pulmonary Tuberculosis (PTB) cases. All suspected and confirmed TB cases are to be reported to Eastern Cape Health facility report of the Electronic Tuberculosis Register (ERT.Net) within a specific time of starting TB treatment. ERT.Net is a record unit for TB patients’ treatment, TB therapy, TB investigation, training and seminars for health care workers and nurses. All TB incidents must be confirmed by medical staff and laboratory tests. 

### 2.3. Ethical Considerations

This research was approved by the Govan Mbeki Research Ethics Committee, University of Fort Hare, Alice, Eastern Cape, South Africa, with reference number-QIN051SAZE01. Also, an approval letter to collect data was obtained from Eastern Cape Department of Health to conduct such research in the province with reference number-EC_2015RP26_384. Any information regarding study subjects used a number instead of their names and was kept confidential. 

### 2.4. Development of the Model

**Figure 2:**
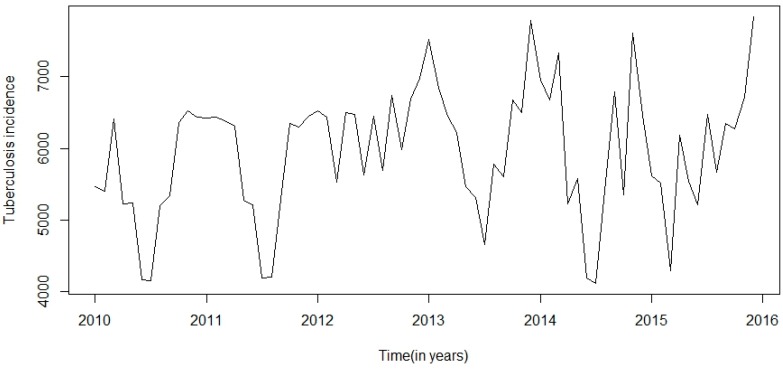
Monthly reported cases of TB prevalence data from 2010 to 2015.

**Figure 3:**
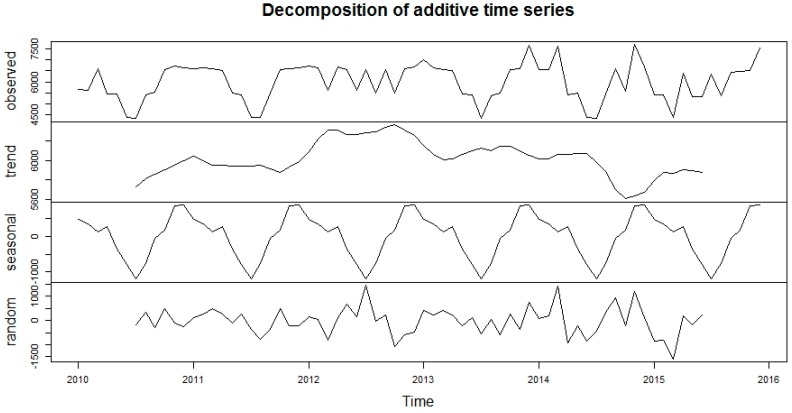
Additive decomposition of monthly time series cases of TB prevalence data.

**Figure 4:**
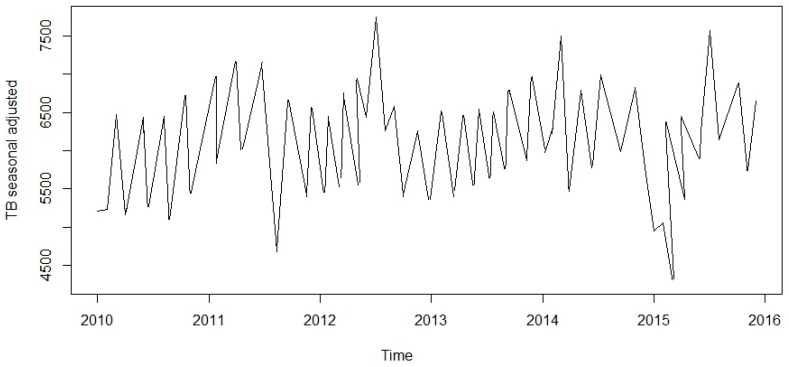
Seasonally adjusted values showing the effects on the monthly reported TB case prevalence.

This research was centred on forecasting comparisons in time series analysis of tuberculosis incidence data. Prior to model fitting, a time series plot was sketched to evaluate the behavioural pattern in the data over a period of years (Figure 2). An additive decomposition of the TB time series was done to describe the seasonality components and trends (Figure 3) and to estimate the seasonal effects that was used to create and present seasonally adjusted values. These adjusted seasonality values are used to remove the seasonal effect so that the trends can be shown clearly (Figure 4). From this graph, we observed that the TB occurrence data had a periodical seasonality movement. Firstly, we looked at ARIMA model to assess the TB data. Moreover, the neural network auto-regression model is mostly used in nonlinear multivariate analysis, which originates outside a system inputs [18] and can be used as a complement of linear analysis. However, the seasonal ARIMA (SARIMA) model and neutral network autoregressive model (NNAR) were used in analysing the trend of the time series data independently of the seasonal components and predicting the monthly TB incidence in South Africa.

### 2.5. Development of the SARIMA Model

Time series seasonality is an unvarying pattern that recurs over *S* periods of time until the pattern changes over again. The SARIMA model integrates both non-seasonality and seasonality factors in a generative model. In the SARIMA model, seasonality in autoregressive (AR) and moving average (MA) terms predict $X_{t}$ using data values and errors at time intervals that are multiples of *S*. The SARIMA model is given as:

(1) $$SARIMA\left(p,d,q\right)\times {\left(P,D,Q\right)}^{S}$$

where *p* = AR order in non-seasonality, *d* = difference in non-seasonality, *q* = MA order in non-seasonality, *P* = AR order in seasonality, *D* = difference in seasonality, *Q* = MA order in seasonality, and *S* = recurrence of time periods in the seasonality pattern. The general SARIMA model has the following form:

(2) $$\Phi \left(B^{S}\right)\varphi \left(B\right)\left(x_{t}-\mu \right)=\Theta \left(B^{S}\right)\theta \left(B\right)\varepsilon_{t}$$

The non-seasonality components are:

(3) $$\begin{array}{l} AR:\varphi \left(B\right)=1-\varphi_{1}B-....-\varphi_{p}B^{p}\\ MA:\theta \left(B\right)=1+\theta_{1}B+....+\theta_{q}B^{q} \end{array}$$

The seasonality components are:

(4) $$\begin{array}{l} AR:\Phi \left(B^{S}\right)=1-\Phi_{1}B^{S}-....-\Phi_{P}B^{PS}\\ MA:\Theta \left(B^{S}\right)=1+\Theta_{1}B^{S}+....+\Theta_{Q}B^{QS} \end{array}$$

In the equations, *B* represents the backward shift operator, $\varepsilon_{t}$ stands for estimated residual error at *t* for μ=0 and $\sigma^{2}$ is constant and $X_{t}$ represents the observed values at *t* (*t =* 1, 2, *…*, *k*), ϕ is a vector of the AR coefficients, θ is a vector of the MA coefficients, Φ is a vector of the seasonal AR coefficients, and Θ is a vector of the seasonal MA coefficients. In the SARIMA model, seasonal subtraction of appropriate order is used to remove non-stationary data from the series. A first order seasonal difference is the deviation between a value and the corresponding value from the previous year and it is expressed as: $x_{t}=y_{t}-y_{t-s}$, for monthly time series (*S*) = 12. Both autocorrelation and partial autocorrelation functions were used to detect six parameters in the components. Akaike information criterion (AIC) and Schwarz Bayesian criterion (BIC) also were performed to verify the better model that fit the data closely. The SARIMA model and SARIMA-NNAR model was built using R software ((R version 3.2.3, Network Theory Ltd., Bristol, UK) with the *auto.arima* () command and *p* value < 0.05 for statistical significance.

### 2.6. Development of the Neural Network Autoregressive (NNAR) Model

Artificial neural network models are widely applied on forecasting methods based on simple mathematical models that allow nonlinear multivariate associations between the dependent variable and its covariates. There are processes of self-organizing and learning in them. The learning rule used to adjust the neutral network weights is based on the function of long and short stochastic dependence of the time series. The approach is tested over six years’ time series data obtained from the TB register and the lag values in the series can be used as variables for a neutral network auto-regression. Feed-forward neutral networks based on the nonlinear autoregressive model for forecasting time series with a layer hidden are considered, and NNAR (*p*, *n*) signifies *p* lag and *n-*nodes to forecast the output $x_{t}$. An NNAR (*p*, *0*) model corresponds to a model of ARIMA (*p*, *0*, *0*) but then without the limitations on the parameters to make sure it is stationary. It is helpful to also add the last observed values in the seasonality data from the same time as inputs. The input of this model is on the learning procedure, which employs the autoregressive neutral network with consideration of stochastic dependence of long or short term values of the time series ($y_{t-1},\;y_{t-2},\;\ldots .,\;y_{t-s})$ used as inputs to forecast the output $y_{t}$ and with *n* neutrons in the hidden layer. 

This is adjusted by a nonlinear function such as a sigmoid, to decrease the effect of excessive input values, in order to make the network robust to outliers. Since the SARIMA model is employed to examine the linear section of the TB data, the residuals part will have non-linear relationships. In the hybrid model, both the linear and nonlinear sections are combined. The estimated occurrence cases of TB at *t* time variable with two input variables were selected from the model.

The model fitting.

### 2.7. Comparison between the Two Models Performance in Simulation

Six parameter indexes were used to compare the goodness of fit efficiency and performances demonstrated with the errors from the two models. The error indexes are mean square error (MSE), Root mean square error (RMSE), mean absolute error (MAE), mean percent error (MPE), mean absolute scaled error (MASE) and mean absolute percentage error (MAPE). They are expressed as follows:

(5) $$\begin{array}{l} MSE=\frac{1}{n}\sum_{t=1}^{n}{\left(X_{t}-{\hat{X}}_{t}\right)}^{2}\\ MAE=\frac{1}{n}\sum_{t=1}^{n}\left|X_{t}-{\hat{X}}_{t}\right|\;RMSE=\sqrt{\frac{\sum_{t=1}^{n}{\left(X_{t}-{\hat{X}}_{t}\right)}^{2}}{n}}\;\\ MAPE=\frac{1}{n}\sum_{t=1}^{n}\frac{\left|X_{t}-{\hat{X}}_{t}\right|}{X_{t}}\;MPE=\frac{100\%}{n}\sum_{i=1}^{n}\frac{A_{t}-F_{t}}{A_{t}} \end{array}$$

where $X_{t}$ = real incidence cases, ${\hat{X}}_{t}$ = estimated incidence and *n* = predictions number, $A_{t}$ = actual value of the quantity being forecast and $F_{t}$ = forecast.

## 3. Results

TB incidence data from January 2010 to December 2015 was used to perform the time series model fit. ACF and PACF plots were used to determine the key parameters (*p*, *P*, *d*, *D*, *q*, *Q*) of SARIMA model. The best model produced from the TB incidence data after the fifth trial was SARIMA (3, 0, 1, 0, 1, 2)_12_ for monthly time series *S* = 12. The model equation is given as: (1 − 0.5112B) (1 − 0.9721B^12^)$X_{t}$ = (1 – 0.7873B^12^) × 20731.651. The estimates and standard error of model parameters and their corresponding significant values are summarised in Table 1.

In the concept of the SARIMA-NNAR model, the NNAR model was verified by using a smoothing constant of α = 0.1 from the range of 0 to 1 for simulation accuracy using *nnetar* and *forecast.nnetar* in a total of repeats networks of each random starting weights are fitted with lagged values of x as inputs and a single hidden layer with size nodes and with this constant, the hybrid model has its lowest MS, RMSE, MAE, MPE, MASE and MAPE. For non-seasonal data, the fitted model is denoted as an NNAR (*p*, *k*) model, where *k* is the number of hidden nodes. For seasonal data, the fitted model is called an NNAR (*p*, *P*, *k*)m model, which is analogous to an ARIMA (*p*, *0, 0*)(*P*, *0, 0*)m model but with nonlinear functions. The NNAR *(p*, *P*, *k*)m model was fitted and forecasted from Exponential triple smoothing (ETS). The values of *p* and *P* were not automatically selected but specified according to the AIC (optimal number of lags). For non-seasonality time series, the default was the best number of intervals (with smallest AIC) for a linear AR (*p*) model. In seasonality, the default values was *P* = 1 and *p* is selected from the best linear model fit to the seasonally adjusted data. These are then averaged when computing forecasts i.e., *k* was specified to *n =* (*p + P +* 0.1)/2 to the nearest integer. 

**Figure 5:**
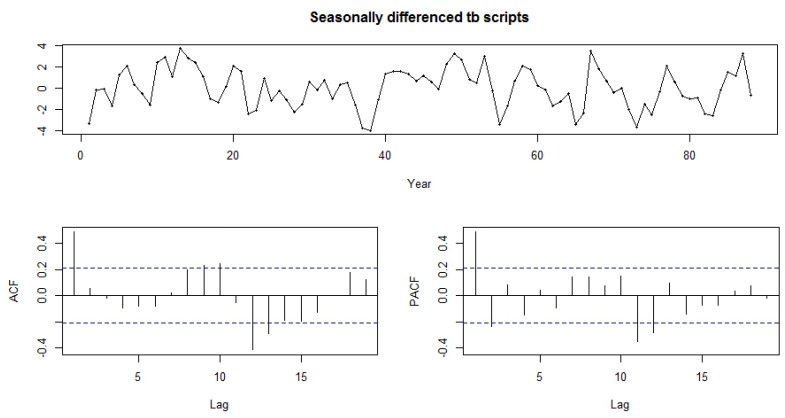
Time plot, ACF and PACF plot for differenced seasonality adjusted monthly TB cases prevalence.

**Figure 6:**
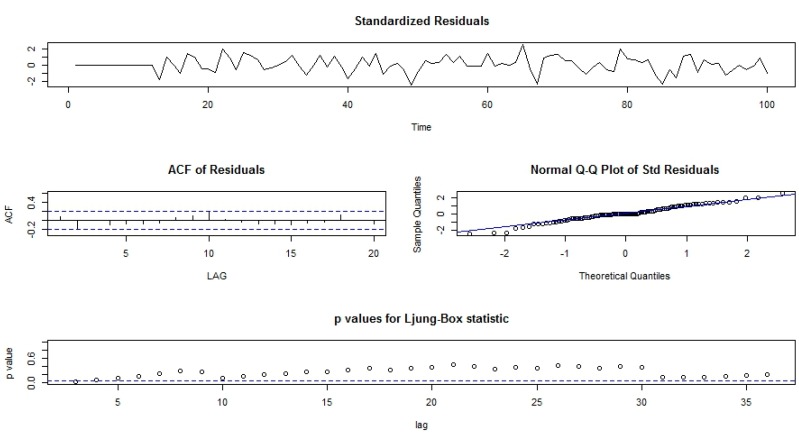
Standardized residuals from the SARIMA model applied to TB prevalence.

Both the functions-ACF and PACF show significant spikes at lag 1 for seasonally differenced data, and almost significant spikes at lag 3 for PACF, showing some added non-seasonality terms to be included in the model (Figure 5). A similar study showed that ACF and PCF of lag 12 show a significant peak suggesting a seasonal component of TB data [19]. The AICc of the SARIMA (3, 0, 1)(0, 1, 2)_12_ model is 327.2, while that for the SARIMA-NNAR (3, 0, 1)(0, 1, 2)_12_ model is 308.31. We attempted other models with AR requisites, but none gives a smaller AICc value. Consequently, we select the ARIMA (3, 0, 1)(0, 1, 2)_12_ model. Its residuals are plotted in Figure 5 and Figure 6. The model passed the residual tests, there are significant spikes in both the ACF and PACF. Entire significant spikes are seen within the significance limits, and the residuals occur to be white noise. A Ljung-Box test shown that the residuals have no outstanding autocorrelations and the model indicated that a Ljung-Box test was “non-significance”, which is desirable. The prediction intervals were accurate due to the non-correlated residuals. Therefore, a seasonality ARIMA model appeared, which passes all the required checks and is ready for prediction.

The two models were compared in predicting the goodness of fit. TB incidence estimations for the forecast accuracy measures of scale-dependent errors on both models are summarised in Table 2. The measures of scale errors in MS, RMSE, MAE, MPE, MASE and MAPE were observed to be lower in the hybrid model compared with the single model. 

**Figure 7:**
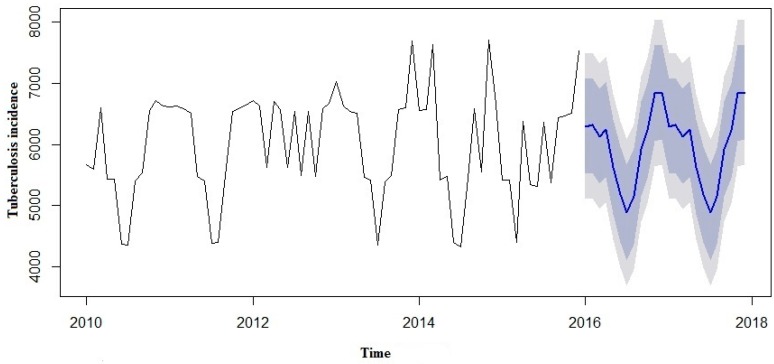
Forecast from SARIMA model applied to TB case prevalence.

**Figure 8:**
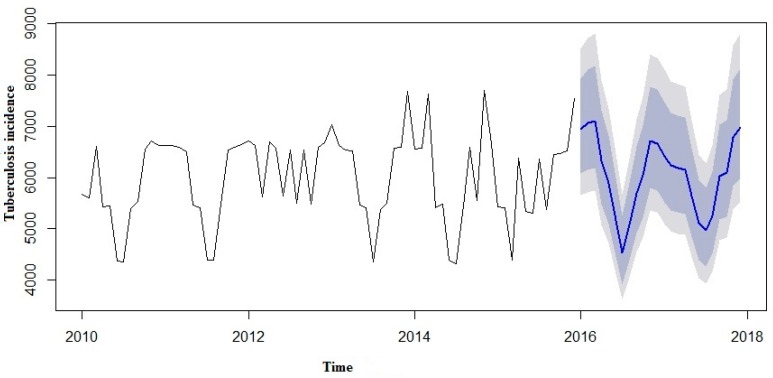
Forecast from SARIMA-NNAR model applied to TB case prevalence.

We made an effort to predict the estimates using both the SARIMA and SARIMA-NNAR models to forecast the number of yearly TB incidence cases in 2016 to 2017 and compare them with the real TB data (Table 3). However, in the forecast model curves (Figure 7 and Figure 8), we observed that TB incidence monthly data in Eastern Cape indicated a marginally increasing trend and a seasonality pattern in the new number of cases of TB incidence. The yearly TB incidence was lower in SARIMA-NNAR in 2016 and 2017 compared to the SARIMA model.

## 4. Discussion

A SARIMA and SARIMA-NNAR model was developed to forecast yearly incidence of TB cases in Eastern Cape. However, in both models, we observed that the time series TB data were simulated well, but the hybrid model that takes into account both the linear and non-linear components performed better than a single model of SARIMA. From our results so far, we could see that the hybrid model of ARIMA and neutral network provide a better forecast with more data characteristics than non-hybrid models. Predictions from the two models are shown in Figure 6 and Figure 7. 

**Figure 9:**
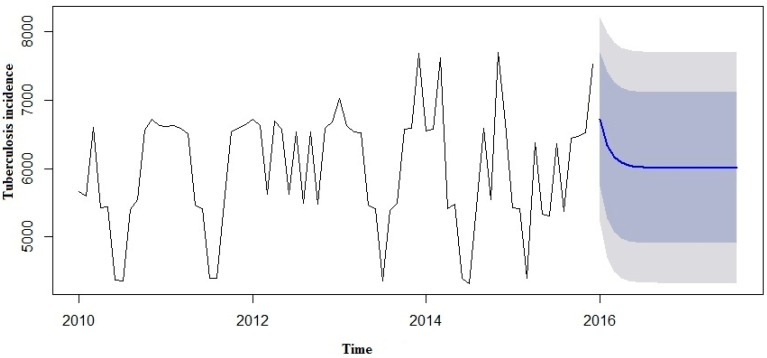
Forecast from ARIMA model with non-zero mean applied to TB case prevalence.

The forecast was noticed to follow the recent trend in the data (this occurs due to subtraction). The rapidly and largely increased prediction intervals indicated that the TB incidence may possibly start increasing or decreasing at any period of time and in a contrast, the point forecasts trend downwards and the prediction intervals allow for the data to trend upwards during the forecast period. This behaviour is different from the one seen in Figure 9 where the prediction intervals are the same for the last few forecast horizons, and the point forecasts are equal to the mean of the data.

In this study, the results observed show that there will be no apparent improvement in the high burden incidence of TB in Eastern Cape in the near future. The predicted outcomes indicated that the reported yearly TB incidence cases will slightly increase in the nearest future in Eastern Cape. The findings revealed that progress in TB control in Eastern Cape needs to be more intensified and adequate interventions are urgently needed.

There was a seasonal variation showing the periodicity of TB incidence in Eastern Cape. The yearly incidence data demonstrate a low incidence in 2010 to 2013 and was higher in 2014 and 2015 respectively. A similar study from the northern part of India showed that the peak period of TB occurrence was observed in April to June and October to December; though the prevalence of TB was lower in other months and there was no noticeable seasonality trends [20]. One of the plausible explanations for this seasonality in Eastern Cape may be the fact that HIV infected people are 30 times more likely to develop active TB due to the effect of HIV/AIDS becoming increasingly apparent, which makes the province to be one of the highest HIV affected areas in South Africa. It is also a low income and underdeveloped province.

Moreover, another notable cause may be the yearly prickly pear festival, one of the most significant yearly festivals in Eastern Cape, which mostly falls in late February or sometimes early March. During the entire month, there are massive crowd movements by various means of transportation. We conjecture that the peak in our study was most likely caused by overcrowding of public transportation over the festival period. 

Another factor enhancing the TB progression in the winter period of the year is low temperature, which forces many people to stay indoors, if the house is poorly ventilated and crowded, this helps in the transmission of TB. A similar study shows that the summer peak was mainly as a result of enhanced winter transmission of TB due to indoor crowding [21]. Another study in United States suggests that reduced winter exposure may not be a strong contributor to TB risk [11,22]. Other possible methods for the seasonality in TB prevalent need to be studied.

Limitations to this study are: firstly, climatic data record (CDR), migration/geographical data and demographic data associated to the target population were not captured in the model fit to show if they constitute a significant cause of TB progression because of data availability limitations like a study conducted in Iran [23]. Secondly, South Africa is a low-middle income country and with differences in geographical entity and climatic conditions, so seasonal variation of TB progression in the various geographic province may be different. Lastly, both models were used only on the data from 2010 to 2015 and verified against only one year of data of TB prevalence. Hence, these results should be interpreted cautiously and should be revisited and analysed with additional time series data using a strong mathematical model.

## 5. Conclusions

Our data confirms that single forecast models can be dealt with by the emergence and application of hybrid models to forecast time series data. The result of the combined model thus, is more effectual and efficient than a single model in generating dependable forecasts of tuberculosis incidence cases. The model indicates that the TB prevalence in Eastern Cape will not increase remarkably in the forthcoming years; it is essential to effect better TB incidence control measures in South Africa. The TB prevalence seasonality from the models also indicate a greater necessity for TB interventions, focused on reducing infectious disease transmission with co-infection with HIV and other concomitant diseases and also on public events and movements during festival periods.

## Figures and Tables

**Table 1:** Estimates and standard error of SARIMA model parameters.

Measurements	Model Terms	Estimates	Standard Error	*t-*Value	*p-*Value
Non-Seasonality	AR1 term	0.5112	0.0930	1.034	0.005
Seasonality	Seasonality AR1	0.9721	0.0091	21.802	0.001
	Seasonality MA1	0.7873	0.1507	2.004	0.014
Coefficient		20731.651	264.521	10.107	0.000

**Table 2:** Prediction accuracy measures of scale-dependent errors on both models.

Models	ME	RMSE	MAE	MPE	MAPE	MASE	AIC	BIC
SARIMA model	0.0408	1.2047	0.9484	106.17	215.51	0.9364	329.02	341.99
SARIMA-NNAR model	0.0095	1.1039	0.7386	92.108	177.62	0.8056	288.56	299.09

**Table 3:** Yearly reported and forecast of TB incidence cases for 2016.

Time	Reported TB Cases	Forecast TB Cases	
		SARIMA Model	SARIMA-NNAR Model
January 2016	5421	6295.522	6103.316
February 2016	5418	6314.305	6122.098
March 2016	4397	6133.734	5941.527
April 2016	6381	6243.660	6051.453
May 2016	5340	5630.462	5438.255
June 2016	5313	5179.841	4987.635
July 2016	6371	4886.305	4694.098
August 2016	5371	5150.119	4957.912
September 2016	6443	5925.772	5733.565
October 2016	6472	6226.831	6034.624
November 2016	6519	6838.240	6646.033
December 2016	7535	6856.255	6664.048
